# The Homes of England


**Published:** 1894-04-21

**Authors:** 


### Studies in Applied Sociology.

### VII.—THE HOMES OF ENGLAND.*

Not the stately homes of England. These, though often not the healthiest in the land, may be left to their owners' care. So may the merchant's villa, fitted, more probably than the ducal mansion, with all the latest devices of sanitary science. But the dwellings of the poor, and those who are not of the poorest—artisans and the humbler middle-class, who cannot supervise the building of their own houses, who cannot afford large rents, who cannot spend guineas in having their drains tested periodically—these are a matter of public concern. Mr. Locke Worthington, who writes on the subject with knowledge and experience, yet indulges in some aspirations which are wholly impracticable. He would fain see all workers live outside, not in our large towns. The idea reminds us of the Frenchman's "Pourquoi ne bat-on pas les villes dans la campagne? L'air y est si pur." A workman's suburb soon ceases to be rural, for apart from the ingrained carelessness as to sanitary matters of the inhabitants, it is impossible to build cottages with the conveniences and air space desirable without making the rent, plus the cost of the journey to and from the town where the bread-winner works, prohibitive to all but the richest of the labouring class. Moreover, the mere lowering of rents, could that be done indefinitely, would not solve the problem. Suppose that, as Mr. Worthington desires, factories and mills were built not in, but beyond, large towns. So they are, generally, to begin with, but the town has the habit of coming up and surrounding them. But suppose an isolated factory, with its little colony of workers. Before long some of these will remove to a town, even though the father risks some loss of wages by so doing. But he has a better chance of finding work for his children, and placing them satisfactorily. The total family income may be increased by the change; even if it is not, the future of the family is better provided for. This, as well as other causes, is helping the depopulation of rural districts. People believe, rightly or wrongly, that there are chances to be had for their children in town which cannot be found in the country, and therefore they seek it. Another reason helps to determine the artisan's preference for town life. He likes to be near his work. We do not speak of the extra cost of travelling implied in living in a suburb, because, under existing conditions, this may be compensated for by better accommodation and a cheaper rent; and the establishment of working men's trains, and possibly, in the future, the nationalisation of railways may bring the expense of travelling to a minimum. But no human rearrangements can affect two factors in the working man's decision—time and fatigue. To live far from his work implies getting up earlier in the morning, and having a shorter rest in the evening. Perhaps the purer air in which he would live should be regarded as compensating for this; but till his education has progressed farther it is doubtful if he will consider this from a scientific point of view. It may be regarded as almost certain that the majority of working men will, either for their own sake or that of their family, prefer to live in town, near their work, and where situations are conveniently obtainable for their children.

It remains, then, to be seen what are the best dwellings the town can provide for them. Judging by appearances, one would pronounce in favour of those rows of two-story cottages which one sees in the artisan districts of many large towns. But nothing would be more unwise. These cottages would indeed be satisfactory if only one family occupied each, but being built for only one household, with conveniences and sanitary arrangements accordingly, they fail completely to meet the wants of the two or four to whom they are sub-let. The rent of a whole house is prohibitively high to all but the best paid wage-earners, and for this no remedy short of a great reconstruction of land laws can be conceived.

There remains then the system of blocks of small flats. Comparatively new in England, this plan, popular in almost every other country, has rapidly gained ground. By means of it better accommodation can be given at the same money, for the same ground-area serves many families. Grave disadvantages there are. Even in those flats which are built for the richer classes complaints of the doings of the piano-player who lives below, and the noisy children who dwell above, are not unknown, and in the smaller flats of the artisan class the vexatious idiosyncrasies of near neighbours are more keenly felt. Any community of property in domestic or sanitary offices is sure to lead to quarrels, accompanied by fierce denunciations of the ancestry and habits of the rival families, and sinister prognostications as to their ultimate destinies—for we have not to deal with a class whose members drop each other gracefully when they agree to differ. Yet, looking to everything, the block plan is undeniably the best. The architecture of such dwellings is of the first importance. Ventilation must be made inevitable, or it will certainly be evaded, and, however little attention be paid to beauty—Mr. Worthington's recommendations as to the decoration of tenements and railway stations seem to us rather a counsel of perfection than a practical advice—the details of sanitation must be carefully carried out. For this reason, among others, the building of such tenements might well be taken up by corporations. It is the interest of every city, while it is not the especial interest of any individual within its bounds, to see that the dwellings of its workers are secure and healthy. A corporation, moreover, is not so intent on large percentages as is the private and often speculative builder. We do not mean, indeed, that the money invested in these buildings should not yield some return—lodgings at the expense of the community are to be given only to paupers or criminals in the places appointed for their abode—but a rent which would give something like the interest on Consols and the necessary sum for repairs, depreciation, and defaulting tenants should suffice. Does that still imply too high a rent? The experience of cities like Glasgow, where the Corporation has built lodgings for the very poor without loss would seem to answer, "No." But in such a case the remedy would seem to lie in such a change in ground rents as would enable a corporation to acquire sites at a price not too far beyond the agricultural value of the land—that, in short, no one man should profit by the growth of a population he has done nothing to bring together, and an industry he has done nothing to develop, but that all who have worked to bring prosperity to any town should thus share in the gain it brings.

* "Dwellings of the People." By T. Locke Worthington, A.R.I.B.A., with an Introduction by G. V. Poore, M.D., F.R.C.P. (London: Swan Sonnenschein and Co. New York: Charles Scribner and Sons. 1893). (Social Science Series).

